# Enzyme Crystals and Hydrogel Composite Membranes as New Active Food Packaging Material

**DOI:** 10.1002/gch2.201700089

**Published:** 2018-01-09

**Authors:** Valentina Mirabelli, Shabnam Majidi Salehi, Luisa Angiolillo, Benny Danilo Belviso, Amalia Conte, Matteo Alessandro Del Nobile, Gianluca Di Profio, Rocco Caliandro

**Affiliations:** ^1^ Institute of Crystallography (IC) National Research Council of Italy (CNR) via G. Amendola 122/o 70126 Bari Italy; ^2^ Department of Economics University of Foggia Largo Papa Giovanni Paolo II, 1 71121 Foggia Italy; ^3^ National Research Council of Italy (CNR) – Institute on Membrane Technology (ITM) via P. Bucci Cubo 17/C 87036 Rende (CS) Italy; ^4^ Department of Agricultural Sciences University of Foggia Food and Environment – Via Napoli 25 71121 Foggia Italy

**Keywords:** antimicrobial food packaging, hydrogel composite membranes (HCMs), hydrogel‐mediated crystallization, lysozyme, protein crystallization

## Abstract

The great antimicrobial and antioxidant potential of enzymes makes them prone to be used as active packaging materials to preserve food from contamination or degradation. Major drawbacks are connected to the use of enzymes freely dispersed in solution, due to reduced protein stability. The immobilization of enzymes on solid supports to create biocatalytic interfaces has instead been proven to increase their stability and efficiency. In this work, it is shown that enzymes crystallized on hydrogel composite membranes (HCMs) can exert an effective antimicrobial action, thus making the composite membrane and crystals biofilm a potential active substrate for food packaging applications. The antimicrobial hen egg white lysozyme is crystallized on the surface of the hydrogel layer of HCMs, and its activity is determined by measuring the decrease in absorbance of *Micrococcus lysodeikticus* culture incubated with the specimen. The overall catalytic efficiency of the antimicrobial HCMs increases by a factor of 2 compared to the pure enzyme dissolved in solution at the same quantity. Because the enzyme in crystalline form is present in higher concentration and purity than in the solution, both its overall catalytic efficiency and antimicrobial action increase. Moreover, the hydrogel environment allows a better protein stabilization and retention during crystals dissolution.

## 1 Introduction

In our age of global sharing and awareness about health and environment, the consumer attention for safer and higher quality foods has increased and so also the demand for solutions to prolong the shelf life of many products, such as dairy, meat, and fresh juices.^1^, ^2^ Indeed, the role of food packaging has become today deeply different from that of a passive object, able to contain foods in whatsoever form; it has turned into an active interface which is asked to interact with the food product and, possibly, with the environment.^3^, ^4^ Regarding the active food packaging, many efforts have been done to develop innovative composite materials enabling to protect food against microbial spoilage,^5^, ^6^ moisture,^7^ or oxidative damages,^8^ and different active compounds have been exploited, such as nanoparticles,^9^ essential oils,^10^ antibiotics, and other organic compounds.^11^ However, for most of them some concerns raise about possible risks for human health, such as heavy metals toxicity, antibiotic resistances, or allergies to some additives.^2^

Enzymes, or biocatalysts, are known since long time in industrial processes as green and safer alternative to chemicals and their immobilization on solid supports in certain cases has been proven to increase the enzyme stability and efficiency at different working conditions.^12^, ^13^ Nowadays they can be also present as functional agent in a packaging material, provided that an efficient immobilization strategy is implemented, and that their catalytic efficiency is not affected by chemical modifications that may occur in covalent binding and cross‐linking procedures.^14^, ^15^ In general, an active compound can be irreversibly bound to the polymeric material, or can be released into the food, depending on the purpose and the immobilization technique adopted. In the former case, it is prevented to migrate into the food, and do not lose its activity by surface interaction with food components, or by dilution below the active concentration level, but it can exert its activity only at the interface between the food and the inner surface of the packaging material, while in the latter case the compound which can diffuse into the food, so extending its range of action, must be safe for the consumer, stable into the food matrix and active even at low concentration.

Lysozyme (E.C. 3.2.1.17) is a single‐peptide protein, which catalyzes the hydrolysis of the β‐1‐4 glycosidic linkage between the *N*‐acetylmuramic acid and *N*‐acetylglucosamine groups found in the peptidoglycan of bacterial cell wall and both its catalytic and lipid‐binding properties are related to the function.^16^ Due to its antimicrobial potential, stability, and harmlessness, it is largely used in biomedical^17^ and food industries, and it has been already attempted to be employed for the development of food packaging materials, by immobilization on polymeric films in the molecular form.^18^, ^19^, ^20^, ^21^, ^22^, ^23^

Protein crystallization allows us to study the structure of the molecule and correlate it to its activity, but it is also a way to purify an enzyme and immobilize it, in order to make it more resistant and effective.^24^ However, crystallized enzymes have been rarely exploited in immobilization techniques, due to the difficulty to stabilize crystals for industrial applications. Indeed, the solution in which crystals grow is usually different from that in which the protein exerts its biocatalysis, and this generates an osmotic stress that disrupts the crystals. Hydrogel materials represent an interesting solution to face this problem, being able to extend the crystal lifespan by preventing osmotic stress.^25^, ^26^ Moreover, the improved efficiency of hydrogel composite membranes (HCMs) as growing media for protein crystals has been recently assessed.^27^ By this approach, protein crystals can be grown directly in the hydrogel environment by means of the porous membrane. The macroporous hydrophobic membrane contacts a feed solution and an extractant (draw solution) on the opposite side. The solvent vaporizes at the feed/membrane interface under a partial pressure gradient established according to the principles of vapor–liquid equilibrium, diffuses through the macropores and condenses at the extractant side. Unique advantages of membrane‐assisted crystallization with respect to conventional crystallizers are the accurate control of the trans‐membrane flux of solvent, that drives the system through tailored and homogeneous supersaturation pathways, and the adjustable chemical–physical properties (surface energy/contact angle) and topography (roughness/porosity/pore size, and size distribution) of the membrane, to modulate the extent of the Gibbs free energy barrier to heterogeneous nucleation.^28^

On these bases, the main aim of this work is to develop an antimicrobial biofilm for active food packaging, by combining the well‐established antimicrobial activity of hen egg white lysozyme (HEWL) along with the crystal‐stabilizing ability of the hydrogel layer in HCMs. By growing lysozyme crystals in previously optimized HCMs, here we realized an active functional composite material where the antimicrobial agent is present in the preserving hydrogel layer and in a pure and concentrated state. Results concerning the antimicrobial activity of lysozyme crystals grown into hydrogel membranes are shown and compared with the solution dispersed enzyme. We demonstrate that the combined effect of immobilized and dispersed enzymes in the early stages of the process enhances the antimicrobial action, producing better results than immobilized enzymes or dispersed enzymes alone. In addition, even if much deal of effort have been directed towards the understanding of the exact interaction mechanism that allows lysozyme to be bound onto membrane surface, the works reported in literature did not fully cover all aspects related to the process. In this study not only a structural investigation of the immobilized lysozyme and an interpretation at the molecular level of the kinetics of the antimicrobial activity have been achieved, but also the implication of an innovative hydrogel stabilizing media for lysozyme crystal improvement retention has been proposed in order to solve the problem of osmotic disruption of crystals.

## 2 Results and Discussion

### 2.1 Enzyme Crystallization

**Figure 1:**
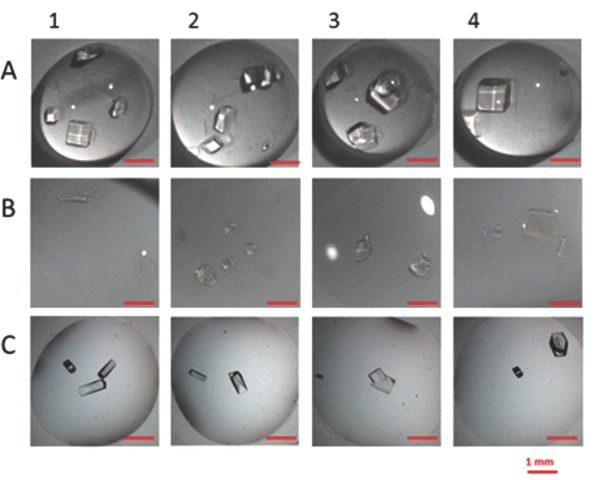
Protein crystallization on different supports. For each crystallization trial, 4 drops have been prepared with 5 µL of a 40 mg mL^−1^ HEWL solution mixed with equal volume of reservoir solution (crystallization condition 1). A) PP; B) PP/PVA‐HCMs; C) on glass.

HEWL has been crystallized by using: (i) pristine commercial polypropylene (PP) membranes sheets, (ii) hydrogel composite membranes made of polyvinyl alcohol (PVA) and poly(ethylene glycol) diglycidyl ether (PEGDE) as cross‐linker, and (iii) conventional glass coverslips, used as templates (**Figure** **1**, lines A, B, and C, respectively). In all cases, crystals have grown after one day and reached their final size in one week, independently on the support or the crystallization conditions used (see below). PP and PVA were chosen because they are not toxic, can be used for food applications, and were proven to be suitable supports for protein crystallization.^27^ PP is an hydrophobic membrane material commonly used for membrane contactors applications^29^ such as membrane distillation and membrane crystallization. Furthermore, PVA is a biocompatible hydrogel material suitable to sustain a well‐hydrated environment for a protein crystal. It has been used in many applications including membrane functionalization.^30^, ^31^

**Figure 2:**
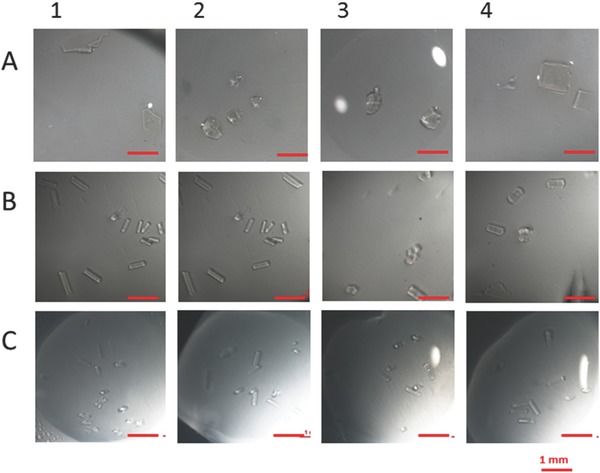
Protein crystallization on PP/PVA‐HCMs at different conditions. For each crystallization trial, 4 drops have been prepared with 5 µL of a A,B) 40 mg mL^−1^ and C) 26 mg mL^−1^ C) HEWL solution mixed with equal volume of reservoir solution. A) Sodium acetate 0.1 m pH 4.6, Sodium chloride 3.5 wt% (crystallization condition 1); B) Sodium acetate 0.1 m pH 4.6, Sodium chloride 7 wt% (crystallization condition 2); C) Sodium acetate 0.5 m pH 4.2, Sodium chloride 5 wt%, PEG 4k 5 wt% (crystallization condition 3).

**Figure** **2** shows crystals grown on hydrogel membranes (PP/PVA–HCMs) at three different crystallization conditions: (1) protein 40 mg mL^−1^, sodium acetate 0.1 m, pH 4.6, NaCl 3.5 wt%; (2) protein 40 mg mL^−1^, sodium acetate 0.1 m, pH 4.6, NaCl 7 wt%;(3) protein 26 mg mL^−1^, sodium acetate 0.5 m, pH 4.2, NaCl 5 wt%, PEG 4000 5 wt% (lines A, B, and C, respectively). It can be noted each crystallization trial differing from the others in crystals yield, size and shape (see Table S1, Supporting Information). In particular, enhanced nucleation rate and reduced crystal growth is observed on PP/PVA–HCMs at crystallization condition 3, compared to conditions 1 and 2.

The possibility to crystallize the lysozyme on the top of PP/PVA–HCMs provides an effective method for enhancing protein stability in the hydrogel environment, thus preventing osmotic shock,^25^ preserving the catalytic activity of the enzyme and allowing its recovery. Furthermore, enzymes immobilized in the crystal form could represent the right compromise between enzyme stability and flexibility of the binding site. In the crystal lattice, in fact, the relative flexibility of protein molecules surrounded by water could easily allow substrates to enter the binding sites, while other immobilization methods, such as covalent linkage or absorption, can block the binding site or reduce too much the enzyme flexibility.^6^, ^32^ Also, in solution the enzyme can be found in different conformations (or folding), both active or inactive, so that only a limited number of solubilized molecules can account for the enzymatic activity. Although in the crystal state a fixed protein conformation is present, the potential partial loss in flexibility is compensated, and potentially overcome, by the increased amount of active molecules in the localized region.

### 2.2 Structural Characterization of Protein Crystals

HEWL crystals grown on PP/PVA–HCMs, PP and standard vapor diffusion were analyzed by X‐ray diffraction at synchrotron light sources and crystallographic parameters are summarized in Table S2 in the Supporting Information. The agreement factor among symmetry‐equivalent reflections, which in ideal condition should have same intensity (Rmerge), and the sigma over noise ratio (*I*/σ) have both their best values for crystals grown on glass and intermediate values for those grown in the presence of PP. Data resolution and the isotropic thermal factor (B Wilson), which are highly correlated parameters describing the degree of coherence of X‐rays diffracted by the atoms in the crystal, increase (worsen) going from standard to PP and HCM‐mediated crystallization. The crystal mosaicity follows the same trend, as already reported in previous studies,^33^ and interpreted as an effect of incorporation of the hydrogel in the crystal lattice during crystal growth.

Although the comparative analysis of structural properties indicates that crystal obtained by HCMs are slightly deteriorated compared to the conventional vapor diffusion, the structural models determined from the X‐ray diffraction data, shown superimposed in Figure S1 in the Supporting Information, have similar agreement with data (Rfree), and negligible structural differences (the root mean square deviation of their Cα atoms is 0.14 ± 0.08 Å). Therefore, the crystal cell parameters (length of axes *a* and *c*) are not affected by the growth support.

A comparative X‐ray diffraction analysis of crystals grown in different conditions on HCMs revealed that diffraction quality, as measured by data resolution, Rmerge, *I*/σ, and B Wilson, is further worsen in crystallization condition 3. In this case, the crystal cell is slightly deformed with respect to crystallization conditions 1 and 2, with a shorter *a* axis.

It can be concluded that no detectable distortion of the structural model is induced by the hydrogel material or by the different crystallization conditions. More interestingly, it is worth noting that the common crystal packing of the HEWL crystals leave their active sites highly exposed to solvent channels, thus preserving the full activity of the enzyme in the solid state. Crystallographic analysis allows estimating that the solvent occupies about 35% of the crystal cell volume.

### 2.3 Antimicrobial Activity at the HCMs–HEWL Crystals Interfaces

**Figure 3:**
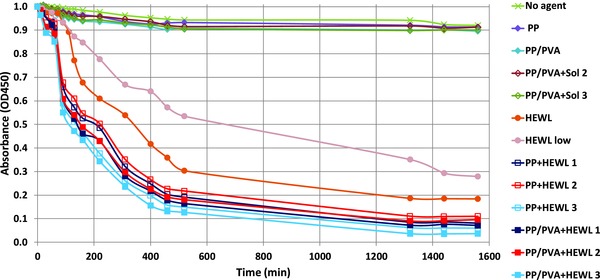
Results of the antimicrobial activity tests given in absorbance at 450 nm (OD450) versus incubation time. HEWL = hen egg white lysozyme; PP = polypropylene membrane; PP/PVA = polypropylene membrane supporting an hydrogel composed of poly(vinyl alcohol) cross‐linked with poly(ethylene glycol) diglycidyl ether. Negative controls: the sole culture medium (no agent), the pristine PP and PP/PVA‐HCMs without crystallization solution, PP and PP/PVA‐HCMs with a drop of crystallizing solution without protein, prepared with the precipitant solution specific of crystallization conditions 2 (PP‐PVA Sol 2) and 3 (PP/PVA Sol 3). Positive controls are HEWL solutions at a protein concentration of 0.1 mg mL^−1^ (HEWL) and at a lower concentration (HEWL low). PP+HEWL and PP/PVA+HEWL are HEWL crystals grown respectively on PP and PP/PVA–HCMs at crystallization conditions 1, 2, and 3.

The catalytic role of lysozyme is well documented in the literature, both in solution and in the crystalline state.^19^, ^20^, ^34^, ^35^ The effect of HEWL in solution and in crystalline form at the interface with HCMs on a culture of *Micrococcus lysodeikticus* is shown in **Figure** **3** as decrease in optical density (OD450). Negative controls (references) consisted in: (i) the sole culture medium (no agent), (ii) pristine PP and PP/PVA–HCMs without crystallization solution, (iii) PP and PP/PVA–HCMs with a drop of crystallizing solution without protein (PP–PVA Sol 2 and PP–PVA Sol 3, prepared with the precipitant solution specific of crystallization conditions 2 and 3, respectively). As expected, OD450 does not reduce significantly for the reference samples, indicating that PP, PP/PVA–HCMs themselves, or the chemicals contained in the crystallizing solution do not have any antimicrobial action, as they do not affect the natural time evolution of the bacterial population. The *M. lysodeikticus* culture is instead heavily affected by the presence of HEWL freely dispersed in solution, as its initial population is halved after 6 or 11 h, depending on the protein concentration. Two positive controls have been used: the first (HEWL) with a protein concentration of 0.1 mg mL^−1^, chosen so that the total amount of protein is the same as in the test with HEWL crystals grown on PP/PVA–HCMs at crystallization conditions 1 and 2, the second (HEWL low) at a lower protein concentration. Notably, HEWL crystals grown on both PP (PP+HEWL) and HCMs (PP/PVA+HEWL) proved to be more effective than HEWL solutions in reducing the vitality of *M. lysodeikticus*, even at the same total amount of protein. In fact, OD450 halves after 135 min for the PP+HEWL material and after 110 min for the PP/PVA+HEWL material in the crystallization condition 3, while in the crystallization conditions 1 and 2 OD450 halves after 215–225 min and after 140–150 min for PP+HEWL and PP/PVA+HEWL, respectively. Interestingly, a better activity for crystals grown on HCMs with respect to those grown on PP alone is systematically envisaged, and an improved antimicrobial efficiency can be ascribed to crystallization condition 3, which appears in both PP+HEWL and PP/PVA+HEWL cases, despite the lower amount of protein used (the crystallization drop contains protein at 26 mg mL^−1^ in condition 3, compared to 40 mg mL^−1^ in conditions 1 and 2). This will be interpreted in the following on the basis of a different crystal yield obtained in condition 3 with respect to conditions 1 and 2.

**Figure 4:**
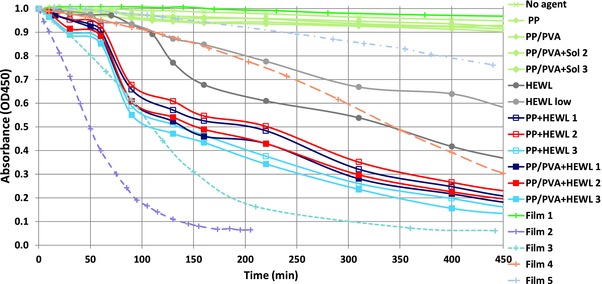
Results of the antimicrobial activity tests shown in a restricted time interval (up to 450 min) and compared with similar tests on films containing lysozyme immobilized in the molecular form in the following quantities: 0 mg (Film 1), 20 mg (Film 2), 50 mg (Film 3), 52.4 mg (Film 4), and 183.4 mg (Film 5).^4^, ^5^

A closer look at the early stage of the process (**Figure** **4**) evidences more complex phenomena occurring when the antimicrobial agent is in the crystal form at the interface with the hydrogel layer of HCMs. Curves corresponding to PP+HEWL and PP/PVA+HEWL cases, in fact, exhibit a common trend, which distinguishes them from HEWL curves. They can be roughly divided in three intervals: a first decrease for the first 60 min, followed by a steady decrease from 60 to 90 min. After 90 min, the decreasing trend is similar to that shown by the protein in solution (HEWL curves). Studies on crystals dissolution rates provide further explanation of such behavior (see further).

To assess the role of the crystalline state in explaining the enhanced antimicrobial action observed for HCMs, results from a previous study on immobilized lysozyme‐based active films have been used.^19^, ^20^ In that case the lysozyme was immobilized in the molecular form on PVA polymer, with glutaraldehyde as bonding agent. Different films were made by using several amounts of lysozyme: 0 mg (Film 1), 20 mg (Film 2), 50 mg (Film 3), 52.4 mg (Film 4), and 183.4 mg (Film 5) (The correspondence with the names adopted in refs. 4, 5 is Film 1 = Film A, Film 2 = Film E, Film 3 = Film D, Film 4 = Film C, Film 5 = Film B.). The lysozyme quantities were measured after film washing; hence they should be referred to the protein actually immobilized on the support. Measurements on antimicrobial activity were made with a similar set‐up as that used for the present study, particularly with the same bacterium–enzyme system and solution volume/active surface ratio (2:1), therefore we can directly compare the absorbance measurements performed on the active films with those performed in this study (Figure 4). It can be noted that OD450 measurements for the film without lysozyme (Film 1) are compatible with our measurements with control samples (composite material without lysozyme); our measurements with lysozyme free in solution are compatible with data from Film 3, while measurements with lysozyme in the crystal form are intermediate between data from Films 3 and 4. Interestingly, the shape of the curves from film samples is similar to that of the lysozyme in solution, and does not show inflection points at 60 and 90 min, which are instead present in the curves related to crystallized lysozyme.

### 2.4 Dissolution Rate Measurements

Solubility tests have been performed with crystals grown on glass, PP, and PP/PVA–HCMs, by using the same buffer as in the antimicrobial activity test, in order to correlate the enzymatic activity with the potential lifespan of protein crystals in solution (see SI). On glass, a single crystal of about 200–500 µm length completely dissolved after around 20–30 min in either 10 or 400 µL of phosphate buffer pH 6.8. At PP and PP/PVA–HCMs interfaces, crystals of different size contained in one drop put in 2 mL of phosphate buffer did not completely dissolve even over 1 h. This highlights that the crystallization by means of the composite hydrogel membranes improves crystal stability and resistance in solution. This effect could be ascribed to the already mentioned incorporation of hydrogel into the crystal lattice. However, it must be considered that the procedure for dissolution rate measurement is quiescent, whereas activity tests were performed instead under continuous stirring. Therefore, crystal dissolution in the latter case is expected to be larger.

An alternative, indirect method to follow crystals dissolution is to measure the increase in protein concentration in the solution over time. Protein concentration measurements under dissolution were performed for crystals grown on PP and PP/PVA–HCMs, for the latter both in quiescent conditions and under stirring (Figure S2, Supporting Information).

In the stirred system, the expected final protein concentration (0.06 mg mL^−1^ for crystals grown under crystallization condition 3) is reached after 60 min; the observed decrease in protein concentration in solution can be ascribed to the absorption of single protein molecules into the hydrogel matrix, in agreement with previous studies.^27^ In quiescent conditions, increases in protein concentration are also detected after 60 min. In this case, the expected final concentration is not reached within the measurement time (160 min), but only after a final stirring stage. It is worth noting that for the stirred case, three repeated measurements give negligible standard deviations all along the incubation time, as expected for a homogeneous solution. On the contrary, for the static system, large fluctuations occur after 60 min, especially for the PP+HEWL system, which point to a nonhomogeneous solution, suggesting a gradual crystal dissolution process, where protein concentration gradients appear.

### 2.5 Antimicrobial Efficacy Determination

**Figure 5:**
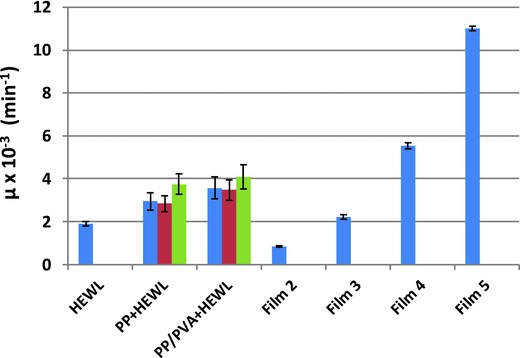
Antimicrobial activity expressed by the parameter μ of Equation (1) estimated by a fitting procedure on experimental absorbance curves. HEWL = hen egg white lysozyme; PP = polypropylene membrane; PP/PVA = polypropylene membrane supporting an hydrogel composed of poly(vinyl alcohol) cross‐linked with poly(ethylene glycol) diglycidyl ether. For PP+HEWL and PP/PVA+HEWL results obtained for crystallization condition 1 (blue bars), 2 (red bars), and 3 (green bars) are reported. Films contain lysozyme in the molecular form in the following quantities: 0 mg (Film 1), 20 mg (Film 2), 50 mg (Film 3), 52.4 mg (Film 4), and 183.4 mg (Film 5).

Experimental absorbance curves can be interpreted in terms of theoretical growth models, as that expressed by Equation (1), allowing the quantification of the antimicrobial activity obtained in each experimental condition. The same least square fitting procedure has been applied to all measurements as well as to those from Conte et al.^19^, ^20^: all the fitted values of the parameters, with their statistical error and related goodness of fit values, are reported in Table S3 in the Supporting Information, while the fitted values for the more relevant parameter, i.e., the maximum decrease rate (μ) are plotted in **Figure** **5**. It can be taken as a measure of the antimicrobial activity and has been compared for all the supports containing the active antimicrobial agent. It can be noted that the antimicrobial activities are much higher for crystallized than for solubilized lysozyme, up to a factor 2 for crystals grown on PP/PVA–HCMs interfaces, and these latter show a statistically significant increase of activity with respect to crystals grown on the PP support. Moreover, it is confirmed that the activities of HCMs supporting crystalline lysozyme are intermediate between those of Films 3 and 4 of refs. 19 and 20.

**Figure 6:**
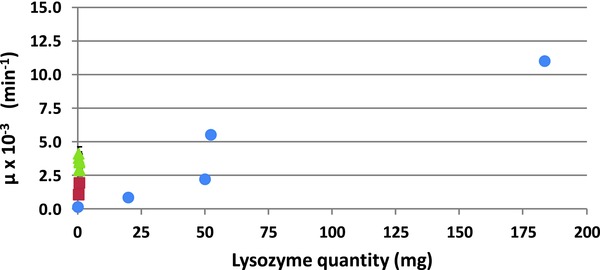
Antimicrobial activity expressed by the parameter μ of Equation (1) estimated by a fitting procedure on experimental absorbance curves plotted versus the quantity of lysozyme present in the test for HEWL in solution (red squares), HEWL immobilized in the molecular form)^4^, ^5^ (blue circles) and HEWL immobilized in the crystal form (green triangles).

To go deeper into this issue, the comparison among activity assessments has been reformulated in terms of quantity of lysozyme present in the experimental setup. For the active films, the quantity refers to the immobilized lysozyme remaining after the film washing, while for the hydrogel membrane composite, the quantity is the sum of the initial quantity of protein inserted in the four drops. The results, shown in **Figure** **6**, make clear the great advantage of using lysozyme in the crystal form on HCMs: it produces a comparable antimicrobial activity with that shown by lysozyme immobilized‐based active films, with a huge protein savings of 0.80 or 0.52 mg for the former (crystallization conditions 1, 2, and 3, respectively) against 50 or 52.4 mg for the latter (Films 3 and 4, respectively). The advantage of using enzyme in the crystal form is even more striking if the comparison is done with the quantity of lysozyme initially added to the films, which is 100 mg for Film 4.

Furthermore, from the analysis of the fitting results (Tables S3 and S4 and Figures S3 and S5, Supporting Information), it can be noted that the fits of absorbance data obtained by using crystalline lysozyme with PP support or PP/PVA–HCMs have a systematically higher χ^2^ than those obtained by using lysozyme dissolved in solution or immobilized on 2D supports.^19^, ^20^ This means that the bacterial mortality occurring in the former cases cannot be adequately described by the standard model used for bacteria mortality rate represented by Equation (1). Indeed, the kinetic of enzymatic activity in crystals is known to be different from that described by the Michaelis–Menten theory for enzymes free in solution, first because the higher protein concentration in the crystal lattice negates the assumption that enzyme should be present in catalytic amount. Also, depending on substrate size, crystals shape and size and protein binding site position into the crystal lattice, the substrate diffusion and the reaction rate range could considerably increase.^36^, ^37^

**Figure 7:**
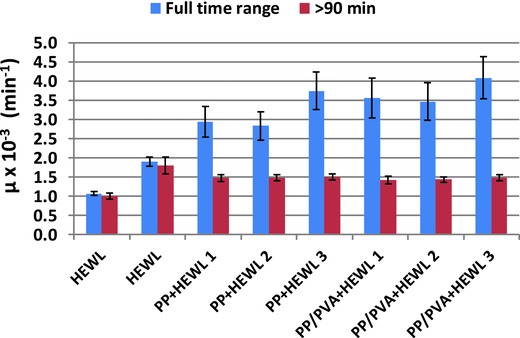
Antimicrobial activity expressed by the parameter μ of Equation (1) estimated by a fitting procedure on experimental absorbance curves applied in the full time range (blue bars) and in a restricted time range (*t* > 90 min) (red bars). HEWL = hen egg white lysozyme; PP =polypropylene membrane; PP/PVA = polypropylene membrane supporting an hydrogel composed of poly(vinyl alcohol) cross‐linked with poly(ethylene glycol) diglycidyl ether.

However, if the fits are repeated for measurements done after 90 min from the beginning of the experiments, this discrepancy is cancelled (Table S4 and Figure S4, Supporting Information). The fitted value of the antimicrobial activity as determined in the full and in the restricted time ranges are plotted in **Figure** **7**: they are comparable for solubilized lysozyme (the same holds for immobilized lysozyme, data not shown), while a sharp decrease of activity is observed for crystallized lysozyme for fitting starting from 90 min. In addition, these data points have much lower fitting errors, indicating a better fit.

Globally, these findings indicate that unique phenomena occur in the first 90 min in the case of lysozyme crystals grown on HCMs, which cannot be described with standard bacterial growth models. Thus, new processes are specific of the lysozyme in the crystal form, which occur within 90 min from the beginning of the exposure to bacteria. After 90 min, these phenomena cease, and the absorbance curves exhibit the same trend as solubilized or immobilized lysozyme. It can be envisaged that the bacterial death process is convoluted with an additional crystal dissolving process, producing an altered kinetics, which should be described by a more complex model.

### 2.6 Antimicrobial Mechanism at the PP/PVA–HCMs–HEWL Crystals Interfaces

**Figure 8:**
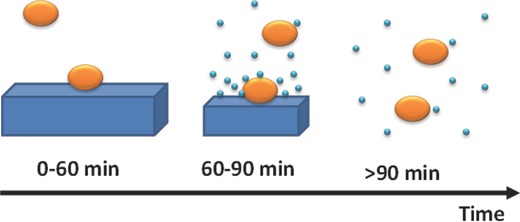
Schematic view of the antimicrobial action of crystallized lysozyme as a function of the incubation time. Bricks represent lysozyme crystals, ellipsoids represent *Micrococcus lysodeikticus* bacteria, and small spheres represent single lysozyme units.

Based on the observed trends of the OD450 curves and evidences on both direct and indirect dissolution rates, we can hypothesize the surface process model outlined in **Figure** **8**. Bacteria interact with enzyme in the crystal form in the first stage of the process (lasting 1 h). Here an improved antimicrobial activity is observed compared to single enzymes immobilized on supports, due to the coordinated action of several protein units in the crystal that come into contact with the bacterium at the same time (the typical size of *M. lysodeikticus* is 0.5–2.0 µm, that of the HEWL crystals is 200–500 µm). After 1 h incubation a mixed antimicrobial process occurs, since the antimicrobial agent is present in the crystal form (with crystals of reduced size due to dissolution) and in solution, with a strong concentration gradient at the crystal/solution interface. The previous bacteria–crystals interaction is summed to the bacteria–single proteins in solution interaction, which is highly efficient due to the large local protein concentration. This explains the steeper decreasing OD450 curves observed between 60 and 90 min of incubation with crystals grown on membrane and hydrogel composite supports. After 1 h and half, all crystals are dissolved. Furthermore, since the stirred condition remove the concentration gradient, the bacteria–enzyme interaction process takes place in the same way as for solubilized protein. Here the OD450 curves strictly follows a Gompertz‐like trend and the resulting activity parameter obtained for the hydrogel composite membranes is comparable to that calculated for the case of free protein in solution at the same initial concentration. A simple statistical model which follows this interpretation is outlined in supporting information.

In this context, the enhanced antimicrobial efficacy observed for HEWL crystals grown in the crystallization condition 3 with respect to those grown under conditions 1 and 2 can be explained in terms of the different yield of crystals. In fact, the larger number of smaller crystals per drop occurred in condition 3 compared to the other two conditions, is likely to increase the probability of contacts between crystals and bacterial cells (given by Equation (S3) in the Supporting Information) while, at the same time, the smaller size of the crystals does not decrease the probability of bacterial cell death for each cell–crystal contact, since crystals are anyway much larger than bacteria.

## 3 Conclusions

Crystallized enzymes at the interface with hydrogel composite membranes are here proposed as new active food packaging material. The proposed solution combines two successful technologies in protein crystallization: membrane‐assisted crystallization and use of hydrogel as growth media to improve crystallization efficiency and crystals resistance to osmotic shock, such as during soaking.

Lysozyme crystals grown in different crystallization conditions showed different antimicrobial efficacies, despite their quite similar crystal lattice and the comparable growth yields. This suggests the occurrence of lysis processes involving bacteria–crystal surface interactions and outlines the possibility of further optimized crystal formation for food packaging.

Results indicate that the antimicrobial efficacy of lysozyme crystals in HCMs is largely improved with respect to enzymes free in solution at comparable quantities, or enzymes immobilized on PVA in the molecular form. In the latter case, the total amount of lysozyme on HCMs is more than 60 times lower than the quantity of lysozyme immobilized in the molecular form necessary to achieve a comparable antimicrobial activity, and more than 125 times lower than the initial quantity of lysozyme on the same active films.

The increased efficiency is observed within 90 min incubation, when bacteria interact with crystallized enzymes, alone or in combination with free enzymes at high local concentration.

PP/PVA–HCMs can thus be envisaged as smart packaging materials, able to release the antimicrobial potential at a fixed time and to modulate its activity, depending on the crystallization and packaging conditions.

## 4 Experimental Section

*Hydrogel Composite Membrane Preparation*: Commercial hydrophobic polypropylene flat sheet membranes were purchased from Membrana Gmbh. PVA (average *M*_w_ = 150 000 g mol^−1^; 98.9% hydrolyzed) and poly(ethylene glycol) diglycidyl ether (average *M*_w_ = 500 g mol^−1^) were obtained from Sigma‐Aldrich, Milan, Italy.

Hydrogel composite membranes were prepared by the solution casting method as follows: PP flat sheet membranes were conditioned by soaking in methanol overnight at room temperature. Thereafter, they were dried between tissue papers. The PVA hydrogel solution 8% (w/v) was prepared by dissolving appropriate amount of PVA in distilled water with heating at 80 °C and constant stirring for 3 h. Then the cross‐linker (PEGDE) 3 wt% respect to PVA was added and mixed. The hydrogel solution was cast on the membrane surface using a manual film applicator adjusted at 50 µm thickness. Then the samples were placed in a thermostatic box at 30 °C to complete the cross‐linking reaction.

*Protein Crystallization*: Hen egg white lysozyme (code L4919), sodium chloride (code 13423), and sodium acetate (code 71183) were purchased from Sigma‐Aldrich, Milan, Italy. PEG 4k was purchased from Hampton Research (code HR2‐605). HEWL was crystallized at 20 °C using the sitting‐drop vapor diffusion technique. PP membrane and PP/PVA hydrogel composite membrane pieces of about 1 cm^2^ were stuck to sitting‐drop bridges (Molecular Dimension, Newmarket, England, UK) on each VDX™ crystallization plate well (Hampton Research, Aliso Viejo, California, USA). A 10 µL drop of crystallization solution was put at the center of each support. Drops were prepared by mixing 5 µL of protein solution with an equal volume of a reservoir solution. Three different crystallization conditions were implemented: 40 mg mL^−1^ protein solution in sodium acetate 0.1 m, pH 4.6 and sodium chloride 3.5 % as precipitant agent, referred to as the crystallization condition 1; 40 mg mL^−1^ protein solution in sodium acetate 0.1 m, pH 4.6 and sodium chloride 7%, as crystallization condition 2; 26 mg mL^−1^ protein solution in sodium acetate 0.5 m pH 4.2 with sodium chloride 5% and poly(ethylene glycol) 4k (PEG 4k) as crystallization condition 3. A HEWL solution was used as positive control in the antimicrobial activity test while PP and PP/PVA–HCM without HEWL were used as negative controls. Each crystallization condition was tested on glass without supports, on PP only and on PP/PVA–HCM, by preparing four drops for each test. Drops were equilibrated against 500 µL reservoir for a few days. Crystal growth was monitored by a Multizoom AZ100 microscope with Digital Sight Camera Control Unit DS‐U, Nikon, in transmission mode for drops on glass, in reflecting mode for drops with membrane supports.

*Crystal Quality Control*: Diffraction properties of enzyme crystals were checked by using the X‐ray beam generated at the European Synchrotron Radiation Facility (ESRF), beamline ID29, and at the Diamond Light Source, beamline I04. Data collections were carried out in cryogenic conditions (100 K), and at beam energies of 11700 eV (ESRF) and 12658 eV (Diamond). The XDS program^38^ was used to perform data reduction, while POINTLESS and AIMLESS programs^39^ were used to find the space group symmetry and scale the diffraction data. The structure was solved by molecular replacement (MR), using the REMO program^40^ included in the package ILMILIONE^41^ with the crystal structure 4N9R^42^ as MR model. The matching between experimental and calculated electron densities was improved by performing an automatic building procedure on the structure obtained by MR in “rebuilt‐in‐place” mode, by using Autobuild wizard included in PHENIX.^43^, ^44^

*Antimicrobial Activity of Crystallized Enzyme*: Lysozyme activity was determined by measuring the decrease in absorbance of a specific microorganism incubated with the tested sample in a buffer solution.^45^ *M. lysodeikticus* (ATCC 4698, Sigma, Milan, Italy) was selected as target Gram‐positive microorganism because of its high susceptibility to HEWL antimicrobial activity. A suspension of lyophilized *M. lysodeikticus* cells was incubated at room temperature in 8 mL of 0.1 m sodium phosphate buffer, pH 6.8, (absorbance at 450 nm) to reach a cell concentration of 10^7^ cells mL^−1^. The obtained cell culture was brought in contact with 4 membrane pieces of about 1 cm^2^ from each crystallization condition. The ratio between solution volume and membrane surface was 2:1. Two HEWL solutions were used as positive control. The first was prepared so that to contain the same amount of protein present in the tests with membrane supports for the crystallization conditions 1 and 2, i.e., 4 (drops) x 40 mg mL^−1^ × 5 µL = 0.8 mg. A 0.1 mg mL^−1^ HEWL solution was then obtained in 8 mL of 0.1 m phosphate buffer at pH 6.8. Between the two different protein concentrations in the crystallization conditions, i.e., 40 and 26 mg mL^−1^, the higher was chosen for this solution because it could be also helpful to highlight the advantage of using a lower protein amount in the crystalline form instead of high amount of solubilized protein. A second HEWL solution (HEWL low) was prepared with a protein concentration lower than 0.1 mg mL^−1^, to test the trend of the absorbance curve in this conditions. The membrane supports without HEWL were used as negative controls, both with and without crystallization reservoir solution. This latter test was done to check if the crystallization solution could serve itself as an additional antimicrobial agent. Cellular lysis was monitored under continuous stirring by the decrease in absorbance at 450 nm (OD450) until a constant value was reached (spectrophotometer UV 1601, Shimadzu model 1642, Shimadzu Europe Ltd., Duisburg, Germany). Each UV measurement was conducted in triplicate. The decrease of absorbance at 450 nm of the culture broth was also checked.

*Dissolution Rate Measurements*: Direct method for estimate crystals lifespan in solution consisted in monitoring under microscope the dissolution of both single crystal on glass, and many crystals in a single drop on PP and PP/PVA–HCM supports. In the first setup, a single HEWL crystal of 200–500 µm length was picked up with a loop from a drop on glass and moved in a new clear drop of 10 µL phosphate buffer, the same used for activity test. Also the dissolution of a single crystal in about 400 µL buffer volume was monitored, seeking the same surface:volume ratio as in the activity tests, in which a total of 20 to 50 crystals per trial were present in 8 mL of buffer. The drop was observed under transmitted light and pictures were taken every 1–5 min with the digital camera until complete dissolution. For crystals on membrane supports, a single drop on the membrane piece was stuck to the bottom of a crystallization plate well and 2 mL buffer were then added slowly and carefully far from the drop. The crystals were observed under reflected light and pictures were taken every 5–10 min.

Indirect method consisted in measuring the lysozyme concentration in buffer, which was expected to increase over the time during the crystals dissolution. The Protein A280 pedestal option in a UV–vis NanoDrop 2000c Spectrophotometer (Thermo Fisher Scientific, Milano, Italy) was used, with default *M*_W_ and ε parameters for lysozyme, already set in the software. Both static and stirred setup were tested: the former was obtained carefully resting one membrane‐supported drop on 2 mL buffer surface, in a crystallization plate well, simultaneously taking the *T*_0_ sample, while the latter was made to reproduce the same setup as in the activity test, i.e., four membrane pieces, all supporting protein crystals, were put into a conical test tube containing 8 mL buffer and then it was kept under continuous stirring.

*Antimicrobial Efficacy Determination*: The average values were calculated from the three values of each UV measurement. Experimental data were fitted by using the Gompertz equation as modified by Zwietering et al.^46^

(1) $$\bar{I}(t)\;=\;K\;+\;Ae^{-e\left\{\frac{\mu e\left(\lambda \;-\;t\right)}{A}\;+\;1\right\}}$$

where $\bar{I}(t)$ is the normalized absorbance at time *t*, obtained by dividing the absorbance measured at time *t* by that measured at *t* = 0, λ can be interpreted as the lag time, and μ as the maximal decrease rate, which can be taken as a measure of the antimicrobial activity. The parameters *K*, *A*, μ, and λ were determined by a least square fitting procedure, implemented as a script of the Root package.^47^

## Conflict of Interest

The authors declare no conflict of interest.

## Supporting information

Supplementary
