# Supplementary material for: Enzyme Crystals and Hydrogel Composite Membranes as New Active Food Packaging Material
Source: Glob Chall. 2018 Jan 9;2(1):1700089. doi: 10.1002/gch2.201700089 (PMC6607345; doi:10.1002/gch2.201700089)
Supplement: Supplementary file 1 — Supplementary [file GCH2-2-1700089-s001.pdf]

Copyright WILEY-VCH Verlag GmbH & Co. KGaA, 69469 Weinheim, Germany, 2018.

Supporting Information

for *Global Challenges,* DOI: 10.1002/gch2.201700089

Enzyme Crystals and Hydrogel Composite Membranes as New Active Food Packaging Material

Valentina Mirabelli, Shabnam Majidi Salehi, Luisa Angiolillo, Benny Danilo Belviso, Amalia Conte, Matteo Alessandro Del Nobile, Gianluca Di Profio, and Rocco Caliandro**

Supporting Information

ENZYME CRYSTALS AND HYDROGEL COMPOSITE MEMBRANES AS NEW ACTIVE FOOD PACKAGING MATERIAL

Valentina Mirabelli, Shabnam Majidi Salehi, Luisa Angiolillo, Benny Danilo Belviso, Amalia Conte, Matteo Alessandro Del Nobile, Gianluca Di Profio, Rocco Caliandro**

Statistical model

In a simple statistical model for bacteria and proteins in solution, the probability P_s of an event of bacterium death is given by the probability of the event that N protein units hit the bacteria in a given time interval. P_s is thus given by $P_s = (p_1)^N$, where p_1 is the probability of a collision of the bacteria with a single protein unit. By following arguments of classical mechanics, it can be written:

$$p_1 = \sigma v n_{prot} \qquad (1)$$

where σ is the cross section for the collision bacteria-protein, n_{prot} is the density of protein units in solution and v is the average bacteria-protein relative velocity, which can be approximated to the average protein velocity v_{prot}. In a pure geometrical view, the cross section can be written in terms of the bacteria (d_{bact}) and protein (d_{prot}) diameters: $\sigma = \frac{\pi(d_{bact} + d_{prot})^2}{4}$, which can be approximated to

$$p_1 = \frac{\pi}{4} d_{bact}{}^2 v_{prot} n_{prot} \qquad (2)$$

since d_{bact}, of the order of 0.5 μm, is much larger than d_{prot}, which is about 0.009 μm. From Eq. (2) it can be noted that p_1 depends on the protein concentration is solution n_{prot}, thus increasing this a consequent increase of bacterium death probability $P_s = (p_1)^N$ is determined.

When instead the protein is present in the crystal form, the probability of an event of bacterium death P_c is given by the probability of the event that the bacterium hit the crystal. In fact, if this event occurs, the bacterium will for sure find N protein units present on the surface of the crystal, since the bacterium (~0.5 μm) is much smaller than the crystal (~500 μm) and the tetragonal lysozyme crystal structure has a surface density of about 10^{12} protein units/μm^2. From mechanics of fluids, it can be written:

$$P_c = \frac{v_{bact} S_{cryst}}{4V} \qquad (3)$$

where v_{bact} is the average velocity of bacteria, S_{cryst} is the total area occupied by protein crystals and V is the total volume of the sample. Thus P_c depends on both the number and shape of crystals present on the support and on flow conditions in the vial, but does not depend on the concentration of the bacteria culture).

When crystals start dissolving, the probability P_{cs} of an event of bacterium death is given by the sum of probabilities P_s and P_c , since both processes occurs at the same time: $P_{cs} = (p_1)^N + P_c$. It should be noted that in this case p_1, which depends from n_{prot} by Eq. (2), is the value corresponding to the local protein concentration near the crystal, which is expected to be higher due to the concentration gradient generated by crystal melting.

Table S1 - Protein crystallization results. Avg refers to crystals present in 4 drops. Size refers to the length of the longer side in parallelepiped or the diagonal in the cuboid shape. Drops were prepared by mixing 5 µL of protein solution with an equal volume of a reservoir solution and equilibrated against 500 µL reservoir for few days. PP=polypropylene membrane; PP/PVA=polypropylene membrane supporting an hydrogel composed of poly(vinyl alcohol) cross-linked with poly(ethylene glycol)diglycidyl ether.

	Crystallization condition 1 (NaCl 3.5%, pH 4.6)	Crystallization condition 2 (NaCl 7%, pH 4.6)	Crystallization condition 3 (NaCl 5% PEG 4k 5% pH 4.2)
Avg number on glass	< 5	≈ 10	> 10
Avg number on PP	< 5	>> 10	nd*
Avg number on PP/PVA	< 5	≈ 10	> 10
Avg size on glass	> 500 µm	< 500 µm	≈ 500 µm
Avg size on PP	> 500 µm	< 500 µm	nd*
Avg size on PP/PVA	≈ 500 µm	> 500 µm	≈ 500 µm
Shape on glass	Parallelepiped/irregular	cuboid	parallelepiped
Shape on PP	Cuboid/irregular	nd**	nd*
Shape on PP/PVA	Plate/irregular	parallelepiped	Parallelepiped/irregular

* Images of crystals grown on PP not available; ** Shape not distinguishable, too many crystals in the drops.

Table S2. Crystallographic parameters of HEWL crystals grown on hydrogel composite membranes (PP/PVA-HCMs) and, for comparison, on PP and in standard vapor diffusion conditions on glass (Glass), on crystallization conditions 1, 2 and 3. Errors are calculated as the standard deviation of determinations from crystals of the same group (whose number is reported on the first row).

	**Glass 2**	**PP 2**	**PP/PVA 2**	**PP/PVA 1**	**PP/PVA 3**
**Number of crystals**	6	7	5	2	3
**Resolution (Å)**	1.2±0.2	1.3±0.3	1.4±0.1	1.26±0.04	1.71±0.05
**Rmerge**	0.04±0.01	0.05±0.02	0.06±0.02	0.19±0.01	0.11±0.02
**I/σ**	22±5	20±3	15±4	2.9±0.6	13±2
**Mosaicity**	0.1±0.1	0.13±0.07	0.3±0.1	0.2±0.1	0.18±0.03
**B wilson (Å^2)**	19±5	17±3	24±3	18.4±0.5	27.8±0.8
**Crystal Cell (Å) a**	78.5±0.4	78.9±0.2	78.4±0.3	78.3±0.5	77.1±0.5
**c**	36.9±0.2	37.00±0.07	37.06±0.08	37.4±0.2	37.5±0.3
**Rfree**	0.25±0.01	0.27±0.08	0.249±0.005	0.27±0.02	0.25±0.01

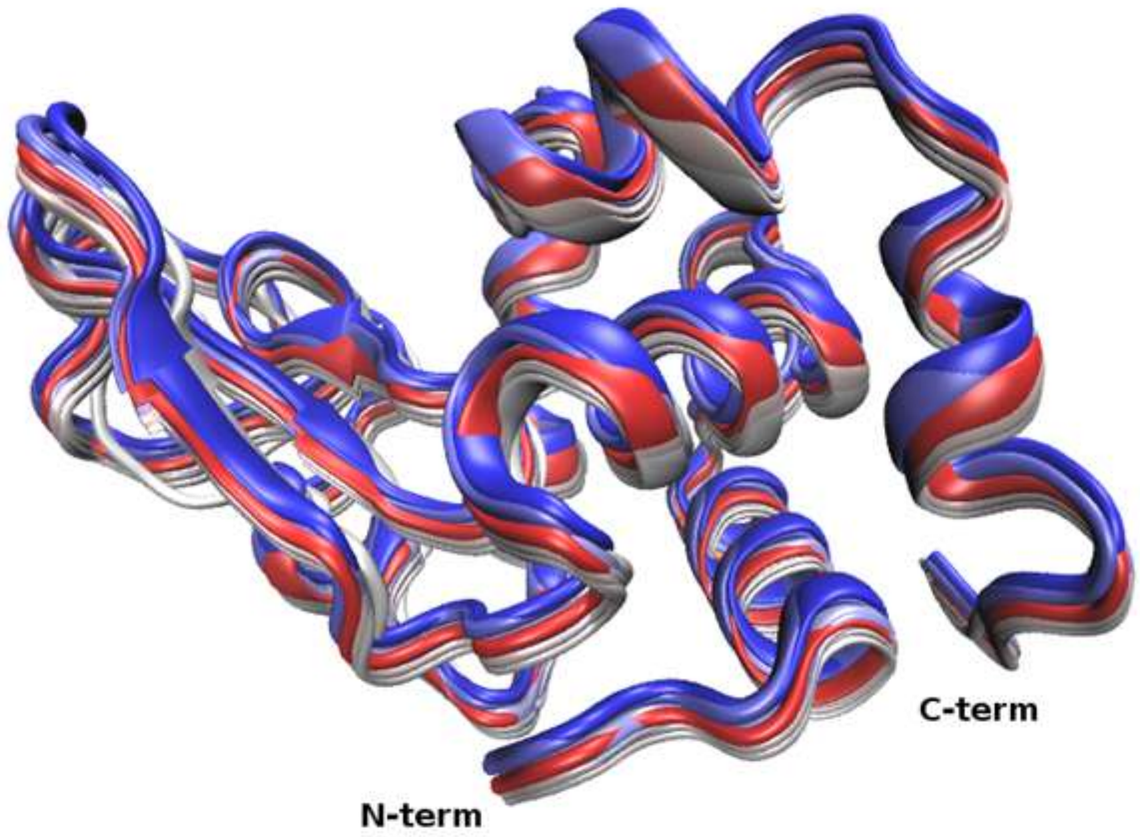

Figure S1. Superposition of structural models of hen egg-white lysozyme (HEWL) determined by crystallographic analysis of 23 crystals grown on different supports and different crystallization conditions.

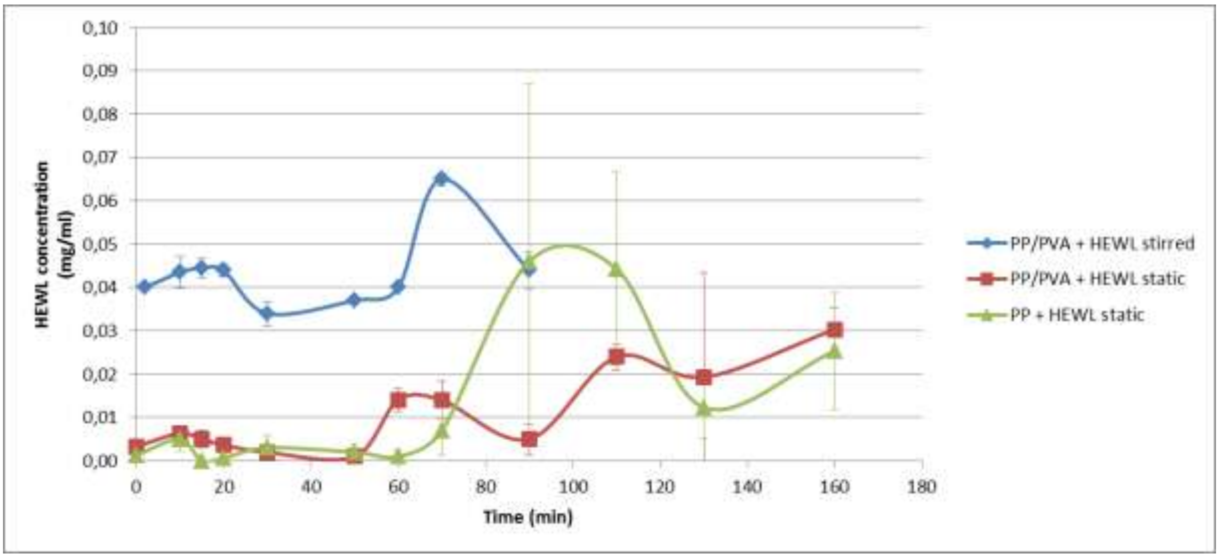

Figure S2 – Results from dissolution rate measurements, i.e. HEWL concentration measurements during crystals incubation in phosphate buffer (see Material and Methods). PP=polypropylene membrane; PP/PVA=polypropylene membrane supporting an hydrogel composed of poly(vinyl alcohol) cross-linked with poly(ethylene glycol)diglycidyl ether; HEWL=hen egg-white lysozyme. For the stirred system, the starting value was not reliable, as during preparation some mechanical stress occurred that dissolved rapidly the crystals, and it was not possible to start the incubation and collect the first sample simultaneously.

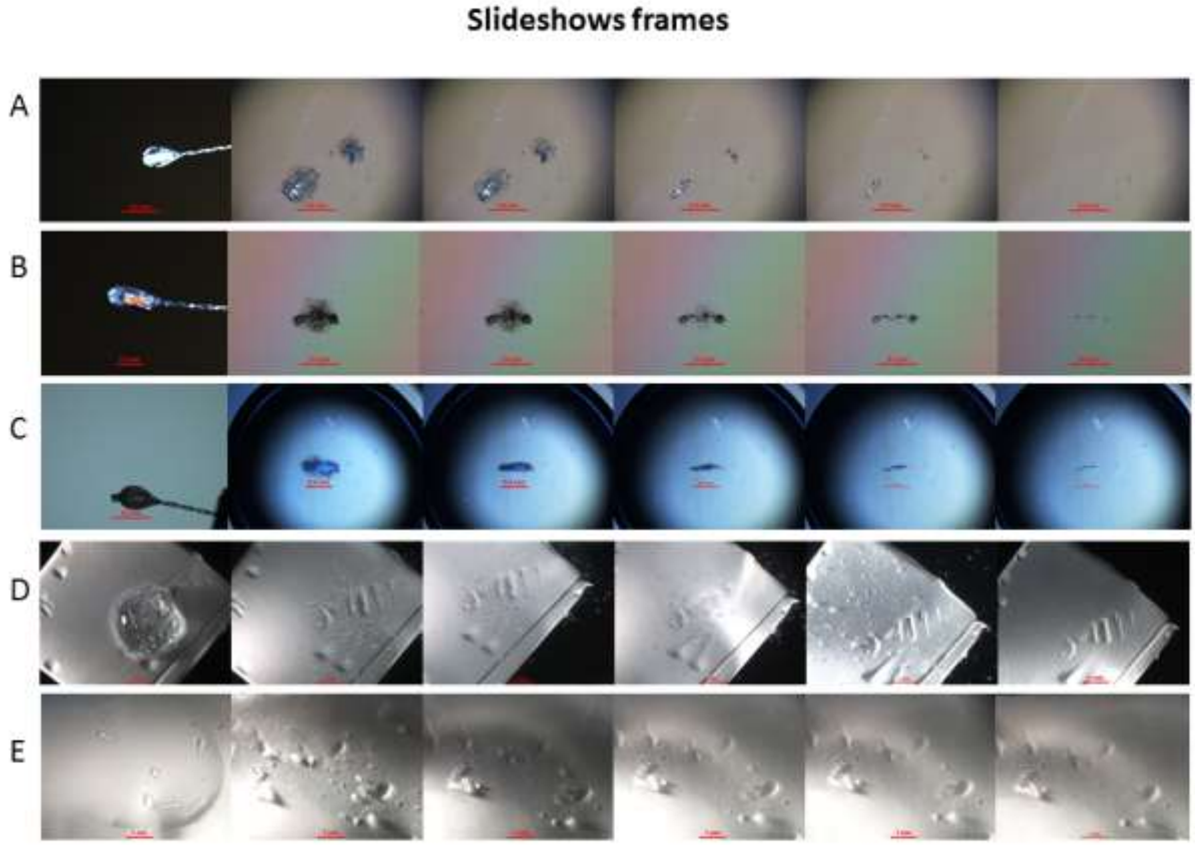

A - single crystal dissolution on glass in 20’
see supporting information file single crystal on glass 20.gif

B - single crystal dissolution on glass in 25’
see supporting information file single crystal on glass 25.gif

C - single crystal dissolution on glass in 30’
see supporting information file single crystal on glass 30.gif

D - crystals dissolution on PP over 1h
see supporting information file crystals on PP.gif

E - crystals dissolution on PP-PVA over 1h
see supporting information file crystals on PP/PVA.gif

Table S3 – Results of the fitting procedure applied to absorbance data points: goodness of fit, represented by the χ^2 value, and fitted values of the parameters included in Eq. (1). No Agent= microbial culture only; HEWL=hen egg-white lysozyme; PP=polypropylene membrane; PP/PVA=polypropylene membrane supporting an hydrogel composed of poly(vinyl alcohol) cross-linked with poly(ethylene glycol)diglycidyl ether. Numbers 1, 2, 3 refers to three different crystallization conditions.

	χ^2 x10^{-2}	*K*	*A*	μ x10^{-3} (min^{-1})	λ (min)
No agent	0.29	1.64±0.04	13.3±0.7	0.12±0.03	4353±2654
HEWL	0.67	1.04±0.01	0.73±0.02	1.80±0.09	17±33411
PP+HEWL 1	4.52	1.04±0.04	0.91±0.05	3.0±0.4	0.0002±416
PP/PVA+HEWL 1	5.11	1.05±0.05	0.93±0.05	3.6±0.5	0.0003±447
PP+HEWL 2	4.13	1.05±0.04	0.90±0.04	2.8±0.4	0.0003±409
PP/PVA+HEWL 2	4.46	1.05±0.04	0.91±0.05	3.5±0.5	0.00003±219
PP+HEWL 3	4.29	1.05±0.04	0.94±0.05	3.8±0.5	0.0003±410
PP/PVA+HEWL 3	4.79	1.05±0.05	0.96±0.05	4.1±0.6	0.0003±431

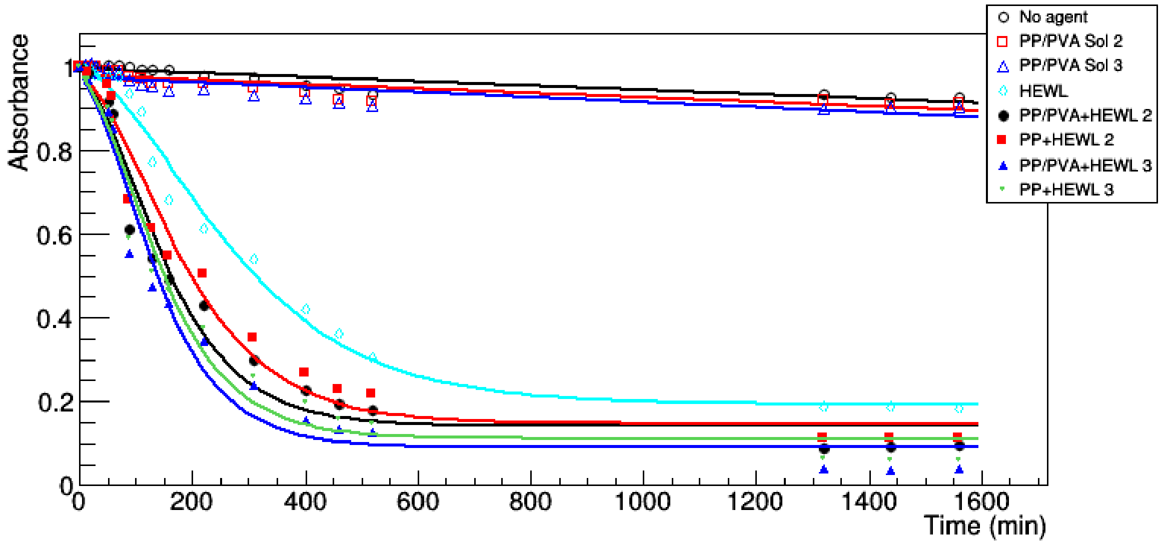

Figure S3 – Results of the fitting procedure applied to absorbance data from selected samples considered in this study. No Agent= microbial culture only; HEWL=hen egg-white lysozyme; Sol=cristallization solution without addiction of protein; PP=polypropylene membrane; PP/PVA=polypropylene membrane supporting an hydrogel composed of poly(vinyl alcohol) cross-linked with poly(ethylene glycol)diglycidyl ether. Numbers 2, 3 refers to three different crystallization conditions.

Table S4 – Results of the fitting procedure applied to absorbance data points taken after 90 min from the starting of the experiment: goodness of fit, represented by the χ^2 value, and fitted values of the parameters included in Eq. (1). No Agent=microbial culture only; HEWL=hen egg-white lysozyme; PP=polypropylene membrane; PP/PVA=polypropylene membrane supporting an hydrogel composed of poly(vinyl alcohol) cross-linked with poly(ethylene glycol)diglycidyl ether. Numbers 1, 2, 3 refers to three different crystallization conditions.

	χ^2 x10^{-2}	*K*	*A*	μ x10^{-3} (min^{-1})	λ (min)
No agent	0.23	1.74±0.05	14±1	0.10±0.06	4095±6565
HEWL	1.64	1.05±0.04	0.85±0.05	1.8±0.2	0.03±846
PP+HEWL 1	0.23	0.78±0.02	0.69±0.02	1.45±0.09	0.00007±116
PP/PVA+HEWL 1	0.26	0.72±0.02	0.64±0.02	1.4±0.1	0.0002±96
PP+HEWL 2	0.17	0.81±0.02	0.70±0.02	1.48±0.08	0.0005±6138
PP/PVA+HEWL 2	0.09	0.73±0.01	0.64±0.01	1.43±0.06	0.16±1681
PP+HEWL 3	0.10	0.71±0.01	0.65±0.01	1.51±0.08	0.0002±63
PP/PVA+HEWL 3	0.11	0.67±0.02	0.63±0.02	1.48±0.08	0.0003±71

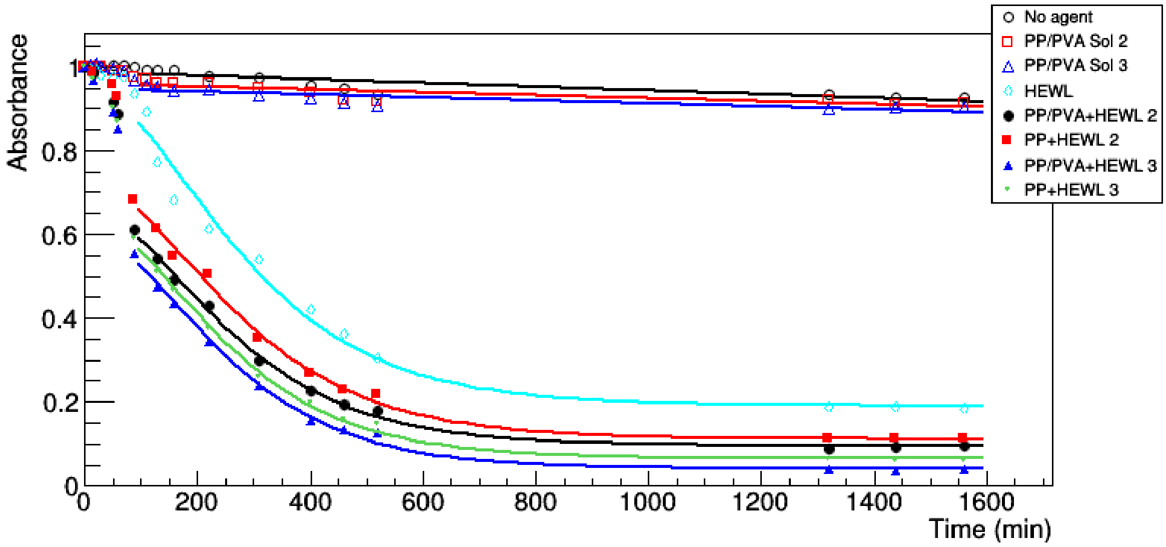

Figure S4 – Results of the fitting procedure applied to absorbance data from selected samples considered in this study. Only data points taken after 90 min from the starting of the experiment are considered for the fitting. No Agent= microbial culture only; HEWL=hen egg-white lysozyme; Sol=cristallization solution without addiction of protein; PP=polypropylene membrane; PP/PVA=polypropylene membrane supporting an hydrogel composed of poly(vinyl alcohol) cross-linked with poly(ethylene glycol)diglycidyl ether. Numbers 2, 3 refers to three different crystallization conditions.

Table S5 – Results of the fitting procedure applied to absorbance data from HEWL immobilized in the molecular form on polymeric films.[4,5]: goodness of fit, represented by the χ^2 value, and fitted values of the parameters included in Eq. (1). The quantity of immobilized lysozyme is: 0 mg (Film 1), 20 mg (Film 2), 50 mg (Film 3), 52.4 mg (Film 4) and 183.4 mg (Film 5).

	χ^2 x10^{-2}	*K*	*A*	μ x10^{-3} (min^{-1})	λ (min)
Film 1	0.02	1.006±0.001	0.05±0.02	0.2±0.2	131±214
Film 2	0.07	1.026±0.008	0.88±0.03	0.84±0.05	119±119
Film 3	1.29	0.97±0.01	0.80±0.02	2.2±0.1	112±15
Film 4	1.04	1.03±0.02	0.97±0.02	5.5±0.1	11±40
Film 5	0.07	1.046±0.004	0.986±0.005	11.1±0.1	0.002±53

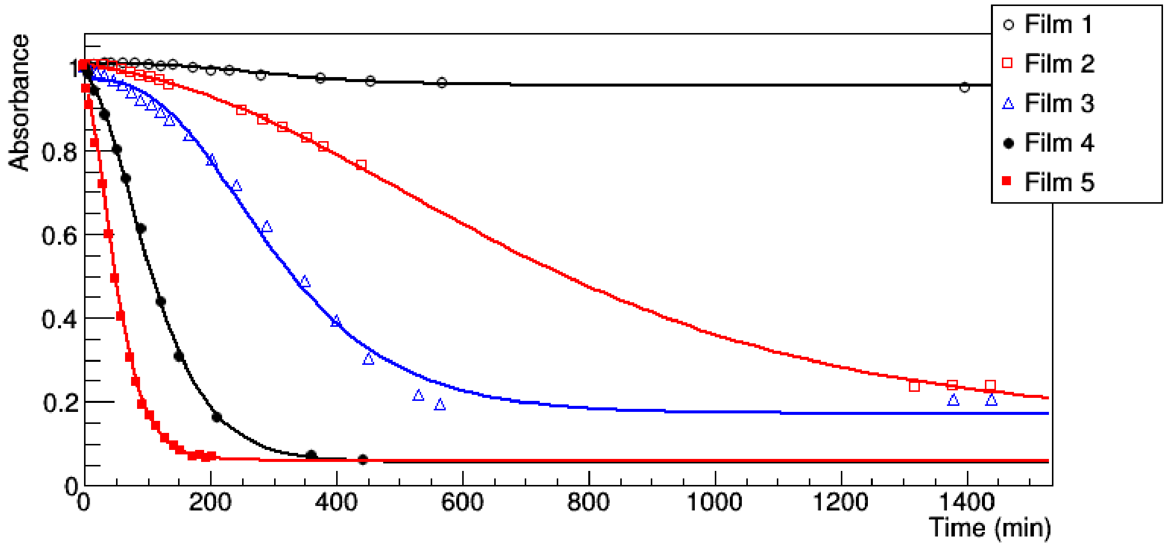

Figure S5 – Results of the fitting procedure applied to absorbance data from HEWL immobilized on polymeric films.[4,5] The quantity of immobilized lysozyme is: 0 mg (Film 1), 20 mg (Film 2), 50 mg (Film 3), 52.4 mg (Film 4) and 183.4 mg (Film 5).
